# Primary Biliary Cirrhosis Is a Generalized Autoimmune Epithelitis

**DOI:** 10.3390/ijms16036432

**Published:** 2015-03-20

**Authors:** Jun Gao, Liang Qiao, Bingyuan Wang

**Affiliations:** 1 Department of Geriatric Gastroenterology, the First Affiliated Hospital of China Medical University, Shenyang 110001, China; E-Mail: gaojunmxr@163.com; 2 Storr Liver Centre, Westmead Millennium Institute for Medical Research, the University of Sydney at Westmead Hospital, Westmead, NSW 2145, Australia; E-Mail: liang.qiao@sydney.edu.au

**Keywords:** primary biliary cirrhosis, biliary epithelial cell, salivary gland epithelial cell, uroepithelium, apoptosis, senescence, mucosal immunity, epithelitis, genetics, epigenetics

## Abstract

Primary biliary cirrhosis (PBC) is a chronic progressive autoimmune cholestatic liver disease characterized by highly specific antimitochondrial antibodies (AMAs) and the specific immune-mediated injury of small intrahepatic bile ducts. Unique apoptotic feature of biliary epithelial cells (BECs) may contribute to apotope presentation to the immune system, causing unique tissue damage in PBC. Perpetuation of inflammation may result in senescence of BECs, contributing to irreversible loss of bile duct. In addition to the classic liver manifestations, focal inflammation and tissue damage are also seen in salivary glands and urinary tract in a significant proportion of PBC patients. These findings provide potent support to the idea that molecular mimicry may be involved in the breakdown of autoimmune tolerance and mucosal immunity may lead to a systematic epithelitis in PBC patients. Thus, PBC is considered a generalized epithelitis in clinical practice.

## 1. Introduction

Primary biliary cirrhosis (PBC) is a chronic cholestatic liver disease characterized by an immune-mediated inflammatory destruction of small intrahepatic bile ducts. Evidence supporting the autoimmune nature of this disorder includes high titers of serum antimitochondrial antibodie (AMA) and autoreactive T cell and B cell responses against mitochondrial self-antigens. AMA is the serological hallmark of PBC with, nearly 95% of PBC patients being positive for this autoantibody. In addition, appearance of AMA prior to the onset of any clinical symptoms usually signals the eventual PBC development. Thus, AMA is considered a highly sensitive and specific biochemical marker for PBC [1].

The natural history of PBC varies considerably across all patients, ranging from an asymptomatic and stable condition with an insidious and slow progression, to a troublesome disorder of repetitious exacerbations and remissions, to an even severe disease of highly symptomatic and rapidly fatal. The progressive destruction of bile ducts in PBC can lead to ductopenia, advanced fibrosis and cirrhosis, and eventually liver failure [2]. Over the past few years, PBC has gained much attention as a topic for extensive research. Although the precise etiology of PBC remains largely obscure, it has been suggested that genetic predisposition, environmental factors and loss of immune tolerance may be of vital importance in driving the development of PBC. Hence, PBC is a multifactorial and a complex disease, just like most other polygenic autoimmune diseases [3,4].

## 2. Primary Biliary Cirrhosis (PBC) Is a Generalized Autoimmune Epithelitis

Several large and comprehensive studies have revealed a strong association between urinary tract infection (UTI) and PBC [5,6]. For example, in an interview-based controlled study of 1032 PBC patients, 59% of female PBC patients claimed to have suffered from recurrent UTI whereas only 52% of the control subjects claimed previous episodes of UTI [5]. Spontaneous remission and relapse are highly prevalent among PBC patients with asymptomatic bacteriuria, and treatment of recurrent bacteriuria was not able to alter the natural history of bacterial infection in PBC patients [6]. On the other hand, female patients with PBC are more prone to develop recurrent UTI compared to the patients with other chronic liver diseases [6]. It is likely that recurrent UTI may increase the mortality rate in PBC patients, although a casual relationship between these two conditions has not been firmly established [6].

A significant number of patients with PBC (47%–73%) show characteristic symptoms of Sjogren syndrome (SS) such as keratoconjunctivitis sicca and xerostomia. Abnormal Schirmer test or diminished salivary flow rate may be found in 30%–50% of PBC patients [7]. Furthermore, salivary gland biopsies have revealed that 26%–93% of PBC patients show the histological damages compatible with SS [7].

The term “autoimmune epithelitis” was first proposed by Moutsopoulos who described the primary SS as a disease characterized by dry mucosa (eyes, mouth) or malfunction of affected parenchymal organs (lungs, kidneys and liver) [8]. It has now been confirmed that PBC can exhibit a progressive immune-mediated epithelial destruction of the biliary tract, salivary gland and urinary tract. Damage of BECs plays a dominant role in the pathologic process of PBC. As such, Gershwin has suggested that the term “autoimmune epithelitis” would accurately reflect the condition for the immune-mediated injury of the target epithelial elements in PBC and SS [9].

## 3. Biliary Epithelial Cells (BECs) Play a Dominant Role in the Development of PBC

### 3.1. Unique Apoptotic Mechanism of BECs May Trigger Selective Tissue Damage in PBC

#### 3.1.1. Inefficient Engulfment of Dead Cells Activates the Immune System

In adult human, billions of cells die every day as a part of the body’s natural processes. Apoptosis is a major type of cell death process that is necessary for the elimination of the dead cells and their harmful debris in order to maintain the tissue homeostasis in multicellular organisms [10]. Apoptotic cells show some characteristic morphological features including cell shrinkage, formation of the membrane blebs, nuclear condensation and fragmentation, and eventually the formation of apoptotic bodies (Abs). A unique feature of the apoptotic cell death is that the membrane integrity of the dead cells is often well maintained [11]. Apoptotic cells are removed either by professional tissue-resident phagocytes (such as macrophages and immature dendritic cells) or by the neighboring non-professional phagocytes and then transferred to lysosomes, where their cellular components are degraded for reuse without release of the potentially harmful inflammatory and immunogenic intracellular contents [10,12].

There is a prevailing belief that impaired clearance of harmful cellular materials by apoptosis contributes to the induction and maintenance of autoimmunity [13,14]. The early recognition and engulfment of apoptotic cells by the phagocytes is necessary for avoiding the damaged cells from entering the late stages of apoptosis, during which cells may no longer be able to maintain their plasma membrane integrity, and therefore can release intracellular contents and provoke post-apoptotic process or secondary necrosis [14]. Late apoptotic cells behave like necrotic cells as they can release potentially inflammatory intracellular contents [15]. Formation of Abs is essential during apoptosis to avoid uncontrolled contact of the immune system with the intracellular autoantigens of the dying cells. Exposure of intact autoantigens within apoptotic vesicles to immune systems may stimulate the innate immunity through secretion of immunomodulatory factors [16,17]. Hence, failure to clear the Abs may play a unique role in the development of the autoimmune disease [15].

#### 3.1.2. Defect in Post Apoptosis Clearance of BECs May Lead to Selective Damage of Small Bile Ducts in PBC

AMA is the highly sensitive and specific serological hallmark of PBC, which specially recognizes liposylated domains within components of the 2-oxoacid dehydrogenase family of enzymes, particularly the E2 component of the pyruvate dehydrogenase complex including PDC-E2, OGDC-E2 and BCOADC-E2. Mitochondrial autoantigens are ubiquitously present in all nucleated cells, however it is not clear why the small intrahepatic bile ducts especially vulnerable to the mitochondrial autoantigen induced immune reaction. Studies have shown that in PBC patients, PDC-E2, OGDC-E2 and BCOADC-E2 remain antigenically active within Abs of the apoptotic BECs and this was largely due to the defect in the clearance of antigenic proteins in BECs, whereas other cells without an altered expression level of autoantigens do not retain the autoantigen epitopes that can be recognized by the AMA in PBC patients [18,19]. It is speculated that depletion of cellular glutathiolation of PDC-E2 may contribute to the preservation of autoantibody recognition [20].

As described in the previous section, BECs can transfer the immunologically intact neoantigens to Abs to form an apotope, which allows the exposure of a potent intracellular autoantigen to the immune system ensuing immunological responses and inflammatory reactions [19]. The apotopes in BECs can be recognized by circulating AMA, leading to an immune complex of AMA-apotope, which may stimulate macrophages to secrete enormous amount of pro-inflammatory cytokines. The subsequent inflammation would lead to increased apoptosis of surrounding cells and BECs, and perpetuation of local inflammation will contribute to chronic damage to biliary tracts [21]. The constant leakage of intact autoantigen may cause antigen accumulation eventually leading to the loss of immune tolerance in PBC.

Antigen-presenting cells (APCs) are able to internalize, process and present immune complex of AMA-apotope, and then activate specific cytotoxic lymphocyte (CTL) to generate pathogenic autoimmune responses [22]. Growing body of evidence has suggested that T cell reaction plays a significant role in immune-mediated cholangitis in PBC. Significantly higher level of PDC-E2-specific autoreactive CD4^+^ T cells and CD8^+^ T cells has been found in liver and regional lymph nodes as opposed to their peripheral counterparts [23].

#### 3.1.3. BECS Are Likely an Active Participant Rather than a Passive Victim in the Development of PBC

It is now well established that BECs are active participants in the initiation and perpetuation of autoimmunity in the pathogenesis of PBC. Extensive data show that activated BECs in PBC can act as non-professional APCs and present pathological epitopes onto their surface. They can also internalize and process Abs from their neighbouring autologous apoptotic BECs as non-professional phagocytes, accompanied by an up-regulation of numerous chemokines and recruitment of more monocytes and macrophages to the inflammatory sites [24]. Chronic recruitment of immune cells may worsen chronic inflammatory infiltration of BECs, leading to progressive bile duct injury.

### 3.2. Senescence of BECs May Contribute to Irreversible Injury of the Bile Ducts in PBC

Bile duct injury and retention of hydrophobic bile acids may lead to further biliary injury. At first, BECs respond to injury by compensative proliferation. Disturbed homeostasis can cause senescence of BECs accompanied by the release of inflammatory cytokines, and thereby contributes to progressive bile duct loss [25]. Cellular senescence is fundamentally a response to a variety of stresses-associated process in which cells irreversibly lose the ability to proliferate [26,27]. Senescent cells secrete many pro-inflammatory cytokines, chemokines, growth factors, matrix remodeling factors, and proteases, collectively termed senescence-associated secretory phenotype (SASP) [28]. SASP possesses several functions including: (1) Autocrine function being to reinforce the development of senescence within the cells of origin; (2) Inflammatory effect provoking activation of innate and adaptive immune system; and (3) Paracrine function being to induce senescence in normal cells surrounding the senescent secretory cells [29]. A limited number of studies support the idea that senescent cells secret factors that are causally linked to degeneration of tissue structure and alteration of tissue function [30,31,32]. SASP consists of a variety of secreted factors that influence cell growth, survival, motility, inflammation, and extracellular matrix remodeling. Importantly, activation of immune cells by SASP can initiate cytolytic responses on senescent cells and neighboring cells [33].

**Figure 1:**
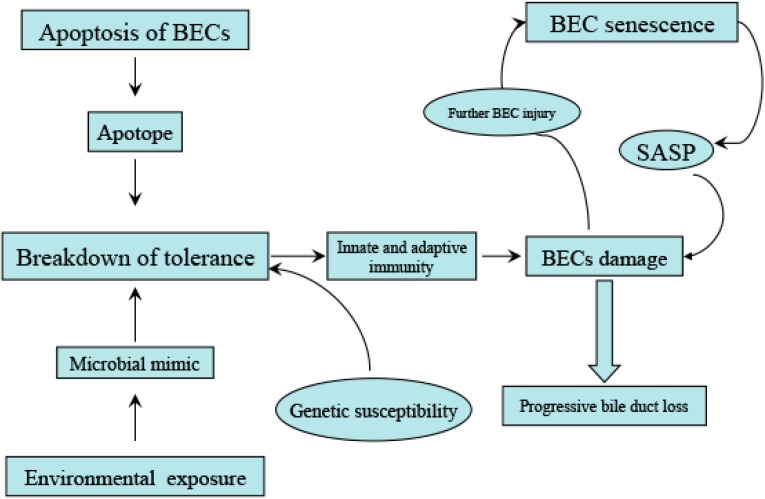
Microbial mimic can trigger the self-destructing immunopathogenic process in primary biliary cirrhosis (PBC). PBC-specific mitochondrial autoantigens remains structurally intact and retains its immunogenicity during biliary epithelial cell (BEC) apoptosis leading to the formation of apotope which can be recognized by circulating anti-mitochondrial autoantibody (AMA). The immune complex would stimulate innate and adaptive immunity in s subject with a susceptible genetic background. Progressive BEC damage tilts the balance between cellular injury and proliferative repair, leading to BEC senescence. The senescent BECs can secret a large amount of senescence-associated secretory phenotype (SASP) which may exert cytolytic response on the senescent cells and neighbouring cells. All of these processes can result in the progressive loss of bile ducts and liver fibrosis.

Senescence of BECs plays an essential role in cellular rejection of liver allografts characterized by progressive bile duct loss. Increased expression of senescence-related p21^WAF1/Cip1^ protein has been observed in BECs during the early phase of chronic liver allograft rejection whereas successful treatment of the allograft rejection led to decreased expression of p21^WAF1/Cip1^ [34]. BECs in the damaged small bile ducts frequently show the histological features of cellular senescence, such as increased activity of SA-β-gal, increased expression of senescence associated p16^INK4a^ and p21^WAF1/CiP1^ [35], as well as the telomere shortening and accumulation of DNA damage [36]. These data indicate that cellular senescence of BECs may be involved in the progressive bile duct loss in PBC and the chronic liver allograft rejection. Further studies have shown that BECs in inflamed and damaged small bile ducts in PBC express significantly higher level of chemokines, such as CCL2 and CX3CL1 which are co-localized with the senescence markers, indicating a possible involvement of SASP in the pathogenesis of PBC [37]. In PBC, the senescent BECs show a very slow or no division despite that they are still metabolically active. Thus, the senescent cells can not be replenished and may squint towards further injury, hence the loss of bile duct in PBC [35]. In addition, as discussed above, senescent BECs can secret SASP which then activate and recruit inflammatory cells to sites of inflammation and modulate the microenvironment, leading to a vicious circle of worsening damage [37] (Figure 1).

## 4. The Role of Other Epithelia in Pathogenesis of PBC

### 4.1. Molecular Mimicry of Urothelium May Trigger Immunological Breakdown

#### 4.1.1. Role of Cross-Reaction between Pathogens and Human Tissues in PBC

Several recent studies have indicated that infectious agents may drive the autoimmunity via molecular mimicry [38,39,40]. This mechanism states that infectious agents may contain an epitope structurally similar to the self-proteins of the infected individuals. Thus, the infections can lead to a cross-recognition of the self-protein and therefore trigger a promiscuous immune response, including both adaptive and innate immune responses [38].

As mentioned above, many well-designed case-control studies have reported a close association between the history of UTI and PBC. *E. coli* is the most prevalent organism isolated in women with UTI. The molecular mimicry between microbial and human PDC-E2 may explain the link between urethral epithelitis and PBC.

Cross-reactivity is not uncommon in PBC because PDC-E2 is a well-conserved sequence with a high degree of identity across all species [41]. The E2 enzymes have a common structure, which consist of an *N*-terminal domain containing a single or multiple lipoyl groups [42]. Although not universally accepted, peptide 163–176 of PDC-E2 (an MHC class II restricted epitope) is identified as the prominent immunodominant epitope. Extensive studies have indicated that the amino acid motif ExDK in the peptide (the amino acid residues E, D, K are at proteins 170, 172 and 173, respectively) is essential for its recognition by specific T cells [43]. The same motif identified in homologous peptides derived from *E. coli* can also be recognized by CD4^+^ T cell clones specific for the PDC-E2^163−176^ peptide [42]. It is reported that the antibody against PDC-E2 of *E. coli* may exhibit 100-fold higher affinity to the same motifs on the biliary epithelia in PBC patients. Intriguingly, lipoyl domains with high degree of similarity to human PDC-E2 has been found in *E. coli*, and such domain has been shown to play an essential role for T cell epitope recognition of PDC-E2 [43].

#### 4.1.2. Role of Toll Like Receptors (TLRs) in Mediating the Immune Response in PBC Patients

Biliary damage in PBC involves innate and adaptive immune mechanisms. It is reported that bacterial pathogen-associated molecular patterns can trigger both innate and adaptive immune reactions through activation of toll like receptors (TLRs) signaling [23]. TLRs are primarily known as the pattern recognition receptor (PRR) for the components of a large number of pathogenic microorganisms, and are considered to be a key component of innate immune signaling. In the initial phase of infection, a rapid inflammatory response may produce numerous chemokines and inflammatory cytokines to block the growth and dissemination of infectious agents. Such adaptive immune responses can quickly initiate T lymphocytes by APC to eliminate infection or damage. Additionally, TLRs play an important role in bridging innate and adaptive immunity [44]. However, over-amplified or dys-regulated TLR signaling may result in inflammation and tissue injury [45,46].

TLRs are expressed in several immune competent cells, such as macrophages, monocytes, and dendritic cells. Recent studies have revealed that both epithelial cells and endothelial cells also express TLRs, and therefore these two types of cells may play an important role in tissue-specific inflammation [47]. TLRs comprise a large family of the pathogen PRR, and TLR4 is the first member of TLR family discovered as a PRR in 1997, and has been shown to play an important role in the recognition of its natural ligand, namely the outer leaflet of the outer membrane of gram-negative bacteria-LPS [48]. Recently, it has been demonstrated that in comparison with other types of liver diseases and healthy controls, patients with PBC frequently show a significantly increased expression level of TLR4 on BECs [49]. *In vitro* studies have shown that the monocytes from the peripheral blood of patients with PBC were hypersensitive to LPS, and LPS could induce BECs to secret inflammatory factors or chemokines such as IL-6, MCP-1 and IL-8. These results have demonstrated that BECs from PBC patients are hypersensitive or less tolerant to LPS exposure [50]. TLR4 may be an important link between the innate immunity and adaptive immunity in patients with PBC.

Further study showed that apotopes of BECs can activate innate immune system via TLR4 signaling, Dys-regulated biliary innate immunity, characterized by hyper-responsiveness to LPS as indicated by higher levels of inflammatory cytokines and chemokine secretion, can lead to the autoimmune injury of BECs [15], and the ensuing inflammation would lead to increased apoptosis of surrounding cells and BECs, and perpetuation of local inflammation contributes to chronic damage of biliary tract [21].

### 4.2. Mucosal Immunity May Induce Systemic Epithelitis of PBC

Human immune system has evolved complex mechanisms to protect the vulnerable epithelial lining of mucous membrane which is constantly exposed to multiple dietary, environmental and microbial antigens. The mucosal surfaces containing high antigenic load are protected by mucosal immunity through production of polymeric antibodies by local plasma cells, mainly polymeric IgA (pIgA). PIgAs can bind to the epithelial polymeric Ig receptor (pIgR) residing on the basolateral surface of mucosal epithelial. The IgA-receptor complex can be excreted through a process termed “transcytosis” after the pIgR is enzymatically cleaved at the mucosal surface. External body fluids such as bile, saliva and urine contain those abundant antibodies [51].

Intense staining of autoantigens at the apical surface of the small BECs lining the bile duct lumen has been observed in PBC patients [52], and AMA is readily detectable in the bile of PBC patients. Furthermore, IgA has been identified as the dominant immunoglobulin isotype against PDC-E2, suggesting a possible involvement of IgA in local immune response during the development of PBC. The immune complex of IgA AMAs and PDC-E2 may enter bile duct cells via a poly-immunoglobulin receptor. The presence of dimeric AMA-IgA in the biliary and mucosal fluids may render the exposed cells more susceptible to apoptosis through persistent transcytosis, resulting in a widening of cell-to-cell junction, and thereby potentially contributes to the pathology of BECs [53]. The titer of anti-PDC-E2-IgA in PBC patients is correlated with increased caspase activation in BECs, making these cells more prone to apoptosis and subsequent bile duct injury [54].

There appears to be a high incidence of focal sialoadenitis, accompanied by lymphoid cell infiltration in patients with PBC, regardless of clinical or histological feature of SS. Moreover, PBC patients showed aberrant apical expression of intense staining of PDC-E2 on the ductual epithelial cells of salivary gland and bile ducts prior to any feature of histological destruction. The staining pattern in both tissues is indistinguishable suggesting that there may be a similar disease process occurring in salivary ducts and BEC [55].

Further study showed that IgA AMA are also actively secreted from epithelial cells and detected in saliva and urine of patients with PBC, in the absence of overt duct injury [56]. In patients with PBC, the AMA present in bile, saliva and urine shows no difference in antigen specificity, epitope recognition and enzyme activity, particularly in the case of secretory type IgA-AMA [57,58]. It is most likely that secretory IgA (sIgA) is derived from local synthesis of pIgA by plasma cells distributed in mucosal tissues [57]. The presence of IgA-anti-PDC-E2 in serum and saliva was associated with the disease progression of PBC patients [59]. IgA AMA detected in secretions might be a more general phenomenon, supporting a notion that PBC might represent a systematic epithelitis [56].

PBC patients showed increased gastrointestinal permeability in both the stomach and the proximal small bowel, compared with patients with other liver diseases and healthy controls [60,61]. It is suggested that altered intestinal barrier function may be involved in chronic bacterial exposure, which may contribute to the stimulation of antibodies that could cross-react with human antigens [60]. On the other hand, evidence showed that there might be a defect in IgA secretion by the intestinal epithelium in PBC patients, which may consequently alter the passage of several antigens into the epithelium [60].

There exists a close anatomical and functional interplay between the gut and liver, namely gut-liver axis. Intestinal bacteria play a pivotal role in the maintenance of gut-liver axis in healthy subjects. It is widely assumed that gut commensal pathogens and intestinal antigens are involved in the pathogenesis of liver injury [62,63]. In order to deal with multitude commensal microbes as well as potential pathogens, the mucosal immune system has evolved specialized functions to identify pernicious pathogens which should be eliminated, whilst providing tolerance to harmless commensal antigens. Nevertheless, microbes may penetrate intestinal defense mechanisms when barrier function of epithelial are damaged and enter the liver through portal circulations. The BECs and gut epithelia share common barrier functions to prevent the spread of infection evading immune surveillance. Intestinal commensals carried to the liver can stimulate immune cells and promote aberrant expression of adhesion molecules and chemokines in liver [64,65,66]. Thus mucosal immune response caused by intestinal commensals may be a precipitating factor in development of PBC.

## 5. Genetics and Epigenetics Mechanisms in Autoimmune Epithelitis

Autoimmune epithelitis in PBC patients is pathogenically connected with genetic and epigenetic abnormalities. A recent study in PBC patients by genome wide association study (GWAS) has identified several susceptibility loci on the host genes involved in innate and acquired immune responses, including IL-12A, IL-12RB2 and STAT-4 [67]. IL-12 signaling has been identified as a key etiologic factor in modulating the disease activity in patients with autoimmune cholangitis and PBC [67]. As mentioned earlier, CD4^+^ T cells play a dominant role in immune-mediated cholangitis in PBC. CD4^+^ helper T cells, classically divided into Th1 and Th2 cells, can regulate immunoreaction to endogenous and exogenous antigens. Th1 and Th2 cells differ from their cytokine production profile in that Th1 cells are mainly involved in the cellular immune reactions whereas Th2 cells are largely involved in the humoral immune reactions. It is reported that anti-IL-12 signaling together with the IL-12 driven IL-γ production promotes Th1 immune response and loss of tolerance by differentiating naïve T cell to Th1 lymphocytes [68]. IL-12A has been linked to celiac disease and multiple sclerosis, whereas STAT-4 is involved in the development of systemic lupus erythematosus (SLE) and rheumatoid arthritis (RA) [19]. These data suggested that multiple genes are shared between immune related diseases. In fact, considerable overlap of genetic susceptibility factors between PBC and several autoimmune disorders has been identified. In this regard, we can speculate that unidentified genetic relationship between PBC and UTI or autoimmune sialadenitis may be present and worth further studying.

Epigenetic modification of the specific X-gene such as DNA methylation may explain the environmental influence on individual’s susceptibility to develop PBC [69]. To support this, female PBC patients with several concomitant autoimmune diseases, such as systemic scleroderma and SS, manifest a skewed X-gene inactivation in their peripheral blood cells [69]. There exists a considerable overlap of genetic susceptibility factors between PBC patients and patients with several other autoimmune disorders. In response to certain environmental stimuli such as infection and toxins, genetically susceptible individuals may develop a particular phenotype or /disease. Indeed, studies have revealed that inter-individual variation in pathogen sensing may result from differences in gene-environment interactions of immune [70].

## 6. Summary

Autoimmune response of BECs plays a dominant role in the development of PBC. Defect in the clearance of post apoptosis protein in the apotope and leakage of intact autoantigen to the immune system leading to constant inflammatory response may be mechanistically responsible for bile duct injury. BECs initially respond to injury by compensative proliferation, while disturbed homeostasis can cause senescence of BECs and release of SASP which may further facilitate cellular senescence in a self-amplifying secretory network and eventually leads to the impaired bile drainage. If persists for a long period of time, loss of small bile ducts, hepatic fibrosis and eventually liver cirrhosis and subsequent liver failure may occur.

**Figure 2:**
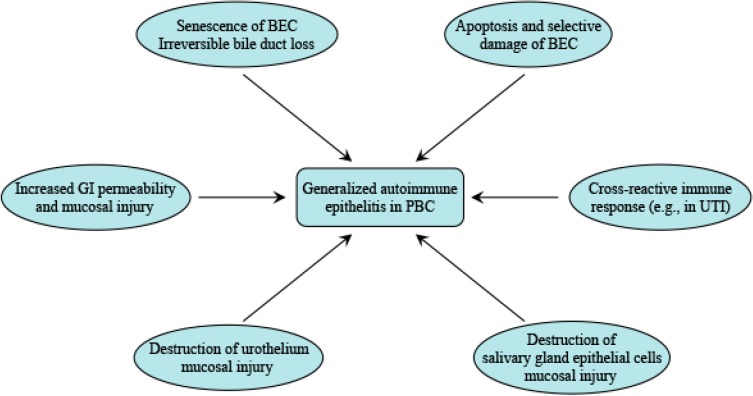
A pathogenetic view of the relationship between autoimmune epithelitis and PBC. (1) PBC-specific mitochondrial autoantigens remains structurally intact and retains its immunogenicity during BECs apoptosis, leading to selective damage of small bile ducts; (2) Progressive BEC damage may lead to BEC senescence and production of SASPs, resulting in progressive loss of bile ducts and liver fibrosis; (3) Infectious agents, such as *E. coli*, may drive urethral epithelitis via molecular mimicry, which may be an initial event in PBC immunological breakdown; (4) IgA AMA may be presents in bile, urine and saliva, supporting the idea that IgA AMA is not restricted to biliary tissues but is a more general phenomenon in PBC; and (5) Mucosal immunity in other ductal epithelial tissues such as salivary glands, urinary tract, and gut may induce generalized epithelitis in PBC.

A strong association of UTI with PBC supports the hypothesis that multiple infectious agents such as *E. coli* may trigger or even exacerbate PBC through molecular mimicry. This phenomenon also explains why many PBC patients suffer from autoimmune uroepithelium injury. IgA mediated immune response and mucosal immunity may be a general phenomenon in patients with PBC and other systematic epithelitis (Figure 2).

The current review on the general autoimmune epithelitis in PBC may provide new insight into the pathogenesis of this disease. More studies on the genetic and epigenetic associations of epithelitis in PBC patients may better reveal the molecular mechanism leading to the development of PBC.

## Abbreviations

AMA: Anti-mitochondrial autoantibody
Ab: Apoptotic body
APC: Antigen-presenting cell
BEC: Biliary epithelial cell
BCOADC-E2: E2 subunit of the branched chain 2-oxo acid dehydrogenase complex
CTL: Cytotoxic lymphocyte
*E. coli*: *Escherichia coli*
LPS: Lipopolysaccharide
OGDC-E2: E2 subunit of the oxo-glutarate dehydrogenase complex
PBC: Primary biliary cirrhosis
PDC-E2: E2 subunit of the pyruvate dehydrogenase complex
pIgA: Polymeric IgA
pIgR: Polymeric Ig receptor
PRR: Pattern recognition receptor
RA: Rheumatoid arthritis
SASP: Senescence-associated secretory phenotype
SS: Sjogren syndrome
SLE: Systemic lupus erythematosus
TLR: Toll like receptor
UTI: Urinary tract infection
